# Draft Genome Sequence of Corynebacterium coyleae UMB8490, Isolated from the Female Urinary Tract

**DOI:** 10.1128/MRA.00393-20

**Published:** 2020-05-14

**Authors:** Abbas Ali Khan, Taylor Miller-Ensminger, Adelina Voukadinova, Alan J. Wolfe, Catherine Putonti

**Affiliations:** a Department of Biology, Loyola University Chicago, Chicago, Illinois, USA; b Bioinformatics Program, Loyola University Chicago, Chicago, Illinois, USA; c Department of Microbiology and Immunology, Stritch School of Medicine, Loyola University Chicago, Maywood, Illinois, USA; d Department of Computer Science, Loyola University Chicago, Chicago, Illinois, USA; Georgia Institute of Technology

## Abstract

Here, we present the draft genome sequence for Corynebacterium coyleae UMB8490, isolated from the catheterized urine of a female with overactive bladder symptoms. The size of the genome is 2,483,223 bp assembled in 62 contigs with an observed GC content of 61.42%.

## ANNOUNCEMENT

*Corynebacterium* is a genus of rod-shaped, Gram-positive, facultative anaerobes (1). The species Corynebacterium coyleae has been associated with urinary tract infections (UTIs), sometimes being the single culture-positive species detected (2). *C. coyleae* has also been associated with urgency urinary incontinence (UUI) and overactive bladder (OAB) (3, 4). *C. coyleae* has also been found in the urine cultures of healthy individuals, but generally in lower abundances, indicating that it may be part of the normal microbiota of the bladder (2, 5). While there is scarce information on the pathogenic potential of this species, it is considered to be an emerging pathogen involved in complicated UTIs as well as nosocomial infections (2, 6).

*C. coyleae* UMB8490 was isolated from a catheterized urine sample obtained from a female patient with OAB as part of a prior institutional review board (IRB)-approved study (University of California, San Diego, IRB no. 170077AW) using the expanded quantitative urinary culture (EQUC) protocol (4). Matrix-assisted laser desorption ionization–time of flight (MALDI-TOF) mass spectrometry was used to identify the genus and species of the isolate, which was stored at −80°C until sequencing. From this freezer stock, *C. coyleae* UMB8490 was first streaked onto a Columbia nalidixic acid (CNA) agar plate and incubated at 35°C with 5% CO_2_ for 24 h. A single colony was selected to inoculate LB broth and grown at 37°C with shaking for 24 h. DNA was extracted using the DNeasy blood and tissue kit following the manufacturer’s protocol for Gram-positive bacteria with the following exceptions: we used 230 μl of lysis buffer (180 μl 20 mM Tris-Cl, 2 mM sodium EDTA, and 1.2% Triton X-100 and 50 μl lysozyme) in step 2 and altered the incubation time in step 5 to 10 min. DNA was quantified using the Qubit fluorometer and sent to the Microbial Genomic Sequencing Center (MiGS) at the University of Pittsburgh for sequencing, where the DNA was enzymatically fragmented using an Illumina tagmentation enzyme. Indices were attached using PCR and sequenced on the Illumina NextSeq 550 platform, producing 1,405,941 pairs of 150-bp reads. Raw reads were trimmed using Sickle v1.33 (https://github.com/najoshi/sickle) and assembled using SPAdes v3.13.0 with the “only-assembler” option for k values of 55, 77, 99, and 127 (7). Genome coverage was calculated using BBMap v38.47 (https://sourceforge.net/projects/bbmap/). PATRIC v3.6.3 and RASTtk were used to annotate the genome sequences (8, 9), and the publicly available genome was annotated using the Prokaryotic Genome Annotation Pipeline (PGAP) v4.11 (10). Unless previously noted, default parameters were used for each software tool.

The *C. coyleae* UMB8490 draft genome is 2,483,223 bp long in 62 contigs with a GC content of 61.42%, genome coverage of 141.47×, and an *N*_50_ score of 92,141 bp. PGAP’s annotation identified 2,290 protein-coding genes along with 51 tRNAs. PATRIC’s annotation also identified a CRISPR array with 120 spacers. While PATRIC identified several genes associated with antibiotic resistance, analysis by ResFinder v3.2 (11) detected only resistance to aminoglycoside, which is used for single-dose treatment of UTIs (12). Further analysis of this genome will advance our understanding of the role that *C. coyleae* plays in the female urinary tract.

### Data availability.

This whole-genome shotgun project has been deposited in GenBank under the accession no. JAAUVV000000000. The version described in this paper is the first version, JAAUVV010000000. The raw sequencing reads have been deposited under the accession no. SRR11441024.
